# The introduction of care robots as a leadership challenge in home care facilities in Finland

**DOI:** 10.1002/nop2.933

**Published:** 2021-06-10

**Authors:** Teemu Rantanen, Teppo Leppälahti, Kirsi Coco

**Affiliations:** ^1^ Laurea University of Applied Sciences Vantaa Finland; ^2^ The Union of Health and Social Care Professionals (Tehy) Helsinki Finland

**Keywords:** attitude, care robot, elderly, home health nursing, leadership

## Abstract

**Aims:**

This paper analyses the factors that influence home care employees’ intention to introduce robots.

**Background:**

The introduction of different kinds of care robots is a topical issue in elderly care now and in the near future.

**Methods:**

Cross‐sectional research conducted through a questionnaire. The survey data (*N* = 162) were collected in five locations around Finland in 2019. The analysis was carried out by regression analysis, Sobel test and by Hayes’ bootstrapping method.

**Results:**

The results show that self‐efficacy is pivotal in the willingness to introduce care robots. Employees’ age increases the enthusiasm to introduce robots but reduces self‐efficacy. Work engagement does not correlate with self‐efficacy or behavioural intention related to the introduction of care robots.

**Conclusions:**

The present paper reveals the significance of attitudes, cognitive factors and age in the adoption of care robots in home care facilities.

**Practical implications:**

It is important to pay attention to supporting the employees’ sense of technology management and the construction of a robot‐positive atmosphere when introducing care robots, and the development of skills of older employees and employees with a lower educational level should be supported.

## 1 INTRODUCTION

Development of high‐tech solutions has been rapid in areas such as medication handling, diagnosis support, operating room operations and telemedicine. Robots for various care tasks have been developed for older people. Bouwhuis (2016) brings out lifting robots, exoskeletons, assistive robots, emotional communication robots and service robots. Robots can provide physical care, social support or medical assistance for older people. Experiences of robots are slightly positive in geriatric health care (Agnihotri & Gaur, 2016).

The use of medicine dispensing robots, advanced security solutions and telecommunication robots has increased. Therapy and social robots have become more common in the care for older people, and many studies have found them to be useful (Agnihotri & Gaur, 2016). However, they are rarely used in private homes. Likewise, various lifting robots (e.g. bear robot RIBA) and wearable robots used in rehabilitation are quite inconvenient to use in private homes. The use of robots to support independent living for older people poses many challenges related to usability and security.

The introduction of care robots creates challenges for the leadership of elderly services. Vichitkraivin and Naenna (2019) have found different resistance factors affecting the adoption of healthcare robot technology. The present paper analyses the significance of employees’ attitudes, self‐efficacy, age, educational level, experience in technology and work engagement, as well as work community norms in terms of the adoption of care robots in homecare facilities. This study is based on Bandura’s (1982) concept of self‐efficacy, Ajzen’s (1991) theory of planned behaviour and focusses on Finnish Registered Nurses, licensed vocational nurses and other health and social care workers.

## 2 BACKGROUND

### 2.1 Cognitive approach to the introduction of care robots

This study approaches issues of nursing leadership from the perspective of the adoption of care robots, employing a framework of cognitive social psychology. Especially, the concept of behavioural intention, which is an individual's willingness to exert effort to perform the target behaviour, plays an important role in models that explain the adoption of healthcare technology (Holden & Karsh, 2010) and care robots (Alaiad & Zhou, 2014; Rantanen et al., 2018; Vichitkraivin & Naenna, 2019). According to the theory of planned behaviour, behavioural intention is affected by self‐efficacy (perceived behavioural control), subjective norms and attitudes (Ajzen, 1991).

Bandura’s (1982) concept of self‐efficacy refers to a person's sense of control over a behaviour. In the context of the adoption of care robotics, the concept represents a person's belief that he/she will learn to use the robots as desired. According to Latikka et al., (2019), robot use self‐efficacy is associated with the acceptance of using robots. There is a significant relationship between nurses’ self‐efficacy and their age, occupation and educational level (Zaki, 2016); however, Lin (2016) noticed that there were no significant differences between groups who were trained and not trained according to items related to self‐efficacy.

A subjective norm is a person's perception of the degree to which other important people approve or disapprove of the target behaviour (Ajzen, 1991; Holden & Karsh, 2010). The relationship of subjective norms and the adoption of care robots have been little studied, but preliminary results suggest an existence of dependence between them (Rantanen et al., 2018). In contrast, in studies related to the acceptance of IT technology in health care, results have been contradictory (Holden & Karsh, 2010).

Nomura et al., (2006) examined negative attitude*s* and fears concerning robots. Attitudes have been studied from the perspective of practical care and the promotion of the independent living of elderly people. In these studies, care robots are considered to be useful in certain tasks in home health care, such as measuring vital signs, setting medication reminders, communication and in case of a fall or other emergency (Alaiad & Zhou, 2014; Broadbent et al., 2012; Mast et al., 2012).

A person's attitude towards his or her own work can also be assumed to be a relevant factor for various innovative development processes. Thus, in this study, a research design based on Bandura´s (1982) concept of self‐efficacy and Ajzen’s (1991) theory of planned behaviour has been complemented by Schaufeli et al.,’s (2006) concept of work engagement which means a positive state of emotion and motivation characterized by vigour, dedication and absorption. This has connections, for example to job performance, innovativeness, health and age (Schaufeli et al., 2006; Hakanen, 2009). In nursing, patient safety requires the engagement of nurses in their practice that emerges from settings of autonomy and results in safer, cost‐effective patient outcomes (Bargagliotti, 2011). Relatedly, there is a positive relationship between age, years of nursing experience and work engagement (Keyko et al., 2016).

### 2.2 Significance of sociodemographic factors

From the perspective of care robot adoption and deployment intentions, certain background variables also play a key role. Flandorfer (2012) stated that the effect of age, gender, technological experience and education is significant in the acceptance of assistive robots. However, studies on the effects of age have been contradictory (cf. Backonja et al., 2017; Flandorfer, 2012; Syrdal et al., 2013). According to Ezer et al., (2009), age did not have a significant effect on the characteristics that participants attributed to their robot, when technology and robot experience were accounted for, and their study suggests younger and older adults with comparable experience with technology will have similar expectations of robots as performance‐oriented machines. According to Kuo et al. (2009), differences related to gender and educational level were shown to be far less important, although people with a lower educational level expressed more negative feelings towards robots than people who had achieved a higher degree of education. Thus, previous studies suggest that certain cognitive factors and factors related to employees’ backgrounds have a significant impact on their willingness to adopt a new technology, which raises the question of how these factors are taken into account in the management of the technological change process.

### 2.3 General perspectives on managing the robot deployment process

Nursing leaders are pivotal when implementing care robots in home care, and it is also crucial to involve employees in the process (De Brún et al., 2019). Jones‐Roberts (2008) noticed that employees engaged in the development of a project appreciated the opportunity to create new approaches. However, when solutions had already been developed by other employees, they did not achieve the same level of commitment or ownership. Nursing leadership needs to conquer obstacles and cope with challenges when facing changes in nursing (Holm & Severinsson, 2014), and nurse leaders need to be aware of the fact that they have an influential role concerning the implementation of new practices and facilitating a willingness to adapt to new technologies (Bianchi et al., 2018). It is, therefore, critical to ensure that employees work at full capacity and with the support of the leadership, who, in turn, are supported by technology (Ganann et al., 2019).

Gifford et al., (2013) found that individual level barriers were related to nurses’ skills and attitudes when implementing new practices. In their study, the perception that there was no reason to change was the second most identified issue related to nurses’ beliefs that implementing the recommendation would not have an influence on patient care or outcomes (Gifford et al., 2013). Maurits et al., (2015) found that employers should focus primarily on the appreciation of nursing staff by senior leadership.

Given that an ageing population poses challenges for aged care, leadership is one important aspect in meeting those challenges (Backman et al., 2017). Thus, nursing leaders should be aware of the different factors which may have impact on employee's willingness to adapt new innovations like care robots, and according to previous research, behavioural intention, self‐efficacy, subjective norm, attitudes, work engagement and sociodemographic factors may influence how employees accept and adapt to change or new innovations.

## 3 METHOD

### 3.1 Aim and hypotheses

This study focusses on Finnish Registered Nurses, licensed vocational nurses and other social and healthcare workers. The aim of the study was to analyse employees’ intentions to introduce care robots in home care.

First, studies based on the theory of planned behaviour (Ajzen, 1991) show that self‐efficacy, attitudes and perceived subjective norm are important factors in the adoption of new health technologies (e.g. Alaiad & Zhou, 2014; Holden & Karsh, 2010). Second, previous studies suggest that age, level of education and experience with technology are important factors in technology acceptance (e.g. Ezer et al., 2009; Kuo et al., 2009 Flandorfer, 2012; Syrdal et al., 2013; Backonja et al., 2017). Third, previous studies show that work engagement is important for innovativeness (Schaufeli et al., 2006; Hakanen, 2009). Based on these results, this study asks How do the factors of self‐efficacy, attitudes, perceived subjective norm, work engagement, age, level of education and experience with technology affect the willingness of home care employees to introduce care robots in their work?

In addition to direct effects, indirect effects related to self‐efficacy are considered. The first hypothesis based on Ajzen´s theory of planned behaviour is.

H1: Home care employees’ behavioural intention depends on self‐efficacy related to the use of care robots, attitudes towards the usefulness of robots in various home care tasks and perceived norms in the work community.

The second and third hypotheses are related to the significance of background variables.

H2: Employees’ age is associated with behavioural intention and self‐efficacy related to the use of care robots in home care.

H3: Employees’ educational level and previous experience with using welfare and health technology are associated with behavioural intention and self‐efficacy related to the use of care robots in home care.

The study further examines the effect of work engagement on behavioural intention and self‐efficacy

H4: The level of employees’ work engagement is associated with behavioural intention and self‐efficacy related to the use of robots in home care.

### 3.2 Settings

The study is a cross‐sectional research, which investigates the adoption of care robots. The research design is based on Bandura´s (1982) concept of self‐efficacy and Ajzen’s (1991) theory of planned behaviour and a previous Finnish study the adoption of care robots (Rantanen et al., 2018). The design has been complemented by the concept of work engagement, and a detailed analysis of background variables (age, educational level and previous experiences with using welfare and health technology) and their mediation effects.

### 3.3 Sample

The study aimed to comprise a comprehensive regional sample. Therefore, municipalities from the Helsinki metropolitan area and other regions in Southern Finland, Eastern Finland, Western Finland and Northern Finland were included in the sample. Adequate regional coverage was achieved by looking at five municipalities (Table 1). One larger city (200,000 inhabitants), two medium‐sized towns (50,000–100,000 inhabitants) and two rural municipalities participated in the study. All home care employees in these municipalities (a total of 1,128 people) were included in the sample. In addition to home visiting employees, Registered Nurses, physiotherapists and other social and healthcare workers working in the offices of home care facilities were included in the sample, as well as supervisors who were involved in client matters. Administrative supervisors and office workers were excluded from the sample.

**TABLE 1:** Respondents (*N* = 162)

Background variable	Value	Sample	Corresponding national value
Age (average)		43 years	41 years^a^
Gender	Women	94%	94%^a^
	Men	6%	6%^a^
Profession	Licensed vocational nurses	71%	70%^b^
	Registered nurses	14%	12%^b^
	Other	15%	18%^b^
Large area	West Finland	25%	25%^c^
	Helsinki‐Uusimaa	21%	30%^c^
	South Finland	31%	21%^c^
	North & East Finland	22%	23%^c^
	Åland	0%	1%^c^

^a^ In Super, which is a large labour union in the healthcare sector in Finland (Finnish and Union of Practical Nurses, 2020

^b^ In home care personnel in Finland (Noro et al., 2015).

^c^ Population in NUTS 2 region (Official Statistics of Finland, 2018).

The total number of respondents was 162, but due to missing values in some of the study variables (Table 2), the analytic sample size ranged from 152–162, depending on the analysis being carried out. The sample size can be considered adequate from the point of view of the research design and the method used. The main method of the study is linear regression analysis, and the number of independent variables is at most seven.

**TABLE 2:** Sum variables

Variable	Items	*n*	mean^a^	*SD*	Cronbach´s α
Behavioural intention	5	162	3.87	0.81	0.872
Attitudes towards care robots	14	158	3.25	0.83	0.910
Perceived behavioural control	6	162	3.76	0.85	0.881
Subjective norm	4	162	2.99	0.80	0.854
Work engagement	9	157	3.90	0.86	0.921

^a^ Scale: 1–5.

### 3.4 Instrument

The questionnaire contained a total of 72 questions. Background questions were related to gender, age, locality, education and experiences with using health and welfare technology. Most of the questions were Likert‐scale statements (1 = totally disagree to 5 = totally agree). Measures related to the concepts of Ajzen (1991) and Bandura’s (1982) theories were based on a study conducted in Finnish home care (Rantanen et al., 2018) (cf. Appendix 1). The measure of behavioural intention included five Likert‐scale items, the measure of self‐efficacy six items and the measure of subjective norm four items. The reliabilities (Cronbach alpha) of all these measures were shown to be quite high in the original study (Behavioural intention: α = 0.875; self‐efficacy: α = 0.901; subjective norm: α = 0.855) (Rantanen et al., 2018).

The questionnaire contained a total of 14 Likert‐scale attitudinal statements about the utility of care robots in various home care tasks (Table 2). The questions were based on an earlier Finnish measure (Rantanen et al., 2017, 2018) and related to promoting safety (four items, α = 0.865 in the original study), practical assistance (four items, α = 0.824 in the original study) and guidance (three items, α = 0.817 in the original study), as well as the relief of anxiety and loneliness. In the present study, two new questions addressing the importance of care robots as a means of increasing the meaningfulness of life and the independence of everyday life were added to the measure.

Work engagement was examined by using the Finnish version (Hakanen, 2009) of Schaufeli et al.,’s (2006) 9 item UWES scale (Utrecht work Engagement Scale). The measure contains nine seven‐step scale questions. The Finnish version of the measure has been used with various groups of employees, and its reliability has been measured as very good (α = 0.91; Hakanen, 2009).

### 3.5 Data collection

The survey data were collected in five locations around Finland in November and December 2019. Initially, home care supervisors were contacted in each municipality and practical arrangements for administering the survey were agreed upon. The information letter and a link to the electronic questionnaire were sent to all municipal home care employees’ email addresses by the supervisors. Employees responded to the questionnaire during their working hours, while they were at the home care facility's office.

### 3.6 Analysis

The data were analysed using SPSS Statistics. First, the data were revised, and two incomplete forms were rejected. The sum variables were formed from the individual items by averaging, and their internal consistency was examined by Cronbach's alpha coefficient.

The normality of the distributions was investigated graphically and by skewness and kurtosis coefficients. The distributions were close to normal. Thus, it was decided to carry out further analyses in a parametric manner. Although the issue is controversial, the use of parametric methods for sum variables based on Likert‐scale items is considered acceptable by many researchers (Clason & Dormody, 1994; Norman, 2010). The distribution of UWES was negatively skewed, and the scales of its items were converted to 5‐point by combining response options “never” and “a few times a year,” as well as options “once a month” and “a few times a month.”

The actual statistical analyses were carried out using the Pearson Product‐moment coefficient and linear regression analysis with the Enter method. Before regression analyses, the normality of the residual distributions and the linearity condition were checked graphically, and the multicollinearity between the independent variables was investigated by the VIF coefficients.

The effect of age was also examined by one‐way analysis of variance, so that the respondents were divided into four age groups. Before the analyses, the assumption of homogeneity of variances was checked by Levene's test. Mediation effect was analysed by the Sobel test.

The significance of previous experience was investigated non‐parametrically using, at first, Spearman's Rho coefficient. For regression analysis, three dummy variables were generated by combining response options 1, 2 and 3 into 0 and options 4 and 5 into 1. The effect of an employee's educational level was analysed by *t* test and by regression analysis using a dummy variable (1 = high education degree, 0 = no high education degree). Indirect effects of previous experience and educational level were analysed by Hayes’ Bootstrapping method. Mediation analysis was made using PROCESS macro 3.3 for SPSS, and 10,000 bootstrap samples were used. The indirect effect was considered significant when the upper and lower bounds (95% confidence intervals) did not contain a value of zero.

## 4 ETHICAL ISSUES

The study was conducted according to the instructions of the The Finnish Advisory Board on Research Integrity (2012) and WMA, 2020. Research approvals were applied for from each municipality that participated in the study. A statement on ethics for the performance and publication of the research was obtained from the FUAS (Federation of Universities of Applied Sciences) Advisory Board on Ethics (1.6.2016).

## 5 RESULTS

### 5.1 Respondents

Respondents’ average age was 43 years, and the majority of respondents (94%) were women. Most respondents (71%) were licensed vocational nurses, and 14% were Registered Nurses. Half of the respondents had worked in home care facilities at least 10 years. The distributions of respondents’ gender, profession and region are close to national values (Table 1).

### 5.2 Sum variables and their reliability

Five sum variables were constructed, and their reliability was good. In this study, for all variables, the Cronbach alpha was over 0.85 (behaviour intention: α = 0.872; attitudes towards care robots α = 0.910; self‐efficacy α = 0.881; subjective norm: α = 0.854; work engagement: α = 0.921) (Table 2).

### 5.3 The effect of self‐efficacy, attitudes and subjective norms

Employees’ behavioural intention to introduce care robots to home care facilities correlates strongly with self‐efficacy, perceived subjective norms and attitudes. These three independent variables based on the theory of planned behaviour have a significant correlation with each other. In contrast, work engagement does not correlate significantly with any other sum variable. A person's age, in turn, relates to self‐efficacy, their subjective norms and work engagement (Table 3).

**TABLE 3:** Pearson correlations (Sum variables, age)

Variable	Behavioural intention	Attitude towards care robots	Self‐efficacy	Subjective norm	Work engagement	Age
Behavioural intention	1					
Attitude towards care robots	0.621	1				
	*p* < .001					
	*N* = 158					
Self‐efficacy	0.773	0.517	1			
	*p* < .001	*p* < .001				
	*N* = 162	*N* = 158				
Subjective norm	0.506	0.343	0.364	1		
	*p* < .001	*p* < .001	*p* < .001			
	*N* = 162	*N* = 158	*N* = 162			
Work engagement	0.033	0.064	0.014	−0.069	1	
	*p* = .685	*p* = .434	*p* = .866	*p* = .392		
	*N* = 157	*N* = 153	*N* = 157	*N* = 157		
Age	0.005	0.014	−0.214	0.170	0.181	1
	*p* = .946	*p* = .861	*p* = .006	*p* = .030	*p* = .023	
	*N* = 162	*N* = 158	*N* = 162	*N* = 162	*N* = 157	

According to regression model 1 (Table 4), self‐efficacy, attitude and subjective norms explain behavioural intention. The effect of self‐efficacy is the strongest, but all these coefficients are significant (*p* < .001). The model explains behavioural intention very well (*R* ^2^ = .70) (Table 4).

**TABLE 4:** Linear regression analysis (Enter). Dependent variable: Behavioural intention

Variable	Model 1 (H1)					Model 2 (H2)					Model 3 (H3a)					Model 4 (H3b)					Model 5 (H4)				
	B	*SE*	*β*	*t*	*p*	B	*SE*	*β*	*t*	*p*	B	*SE*	*β*	*t*	*p*	B	*SE*	*β*	*t*	*p*	B	*SE*	*β*	*t*	*p*
(constant)	0.357	0.192		1.87	.064	0.072	0.235		0.307	.759	0.130	0.239		0.543	.588	0.130	0.241		0.537	.592	−0.033	0.274		−0.122	.903
Attitude	0.252	0.052	0.256	4.84	<.001	0.241	0.052	0.245	4.65	<.001	0.222	0.053	0.226	4.18	<.001	0.243	0.052	0.246	4.64	<.001	0.242	0.052	0.244	4.69	<.001
Self‐effic.	0.540	0.051	0.565	10.7	<.001	0.574	0.053	0.600	10.9	<.001	0.563	0.053	0.590	10.6	<.001	0.560	0.055	0.587	10.1	<.001	0.605	0.053	0.627	11.3	<.001
Subj. norm	0.220	0.050	0.212	4.41	<.001	0.194	0.051	0.187	3.80	<.001	0.198	0.052	0.191	3.83	<.001	0.192	0.052	0.185	3.71	<.001	0.167	0.053	0.159	3.17	.002
Age						0.006	0.003	0.097	2.06	.041	0.006	0.003	0.099	2.09	.039	0.006	0.003	0.095	2.00	.047	0.007	0.003	0.112	2.32	.021
High educ.^a^											0.130	0.092	0.065	1.41	.161										
Experience^a^																0.008	0.096	0.004	0.080	0.937					
Exp(good^a^																0.086	0.093	0.053	0.92	0.358					
Exp.(bad)^a^																−0.132	0.080	−0.076	−1.65	0.102					
Work eng.																					0.000	0.042	0.000	0.007	0.994
*R* ^2^/ adj. *R* ^2^	0.699/0.693					0.707/0.699					0.708/0.698					0.712/0.698					0.720/0.711				
*F*	119.0 (*p* < .001)					92.2 (*p* < .001)					72.2 (*p* < .001)					52.3 (*p* < .001)					75.8 (*p* < .001)				

^a^ Dummy variable.

### 5.4 Significance of age

Behavioural intention and age do not correlate significantly, *r*(160) = .005, *p* = .946, and there is no significant difference in behavioural intention between different age groups, *F*(3, 158) = 0.087, *p* = .967. In contrast, age and self‐efficacy correlate significantly with each other *r*(160) = −0.214, *p* = .006), and there are significant differences between age groups in self‐efficacy (*F*(3, 158) = 3.41, *p* = .019). According to regression model 2 (Table 4), the effect of employees’ age is significant. Furthermore, according to regression model 1 in Table 5, the effect of age on self‐efficacy is significant.

**TABLE 5:** Linear regression analysis (Enter). Dependent variable: Self‐efficacy

Variable	Model 1 (H2)					Model 2 (H3a)					Model 3 (H3b)					Model 4 (H4)					Model 5				
	B	*SE*	*β*	*t*	*p*	B	*SE*	*β*	*t*	*p*	B	*SE*	*β*	*t*	*p*	B	*SE*	*β*	*t*	*p*	B	*SE*	*β*	*t*	*p*
(constant)	4.38	0.235		18.6	<.001	4.27	0.236		18.1	<.001	4.055	0.235		17.3	<.001	4.24	0.358		11.8	<.001	3.96	0.231		17.1	<.001
Age	−0.014	0.005	−0.214	2.77	.006	−0.014	0.005	−0.205	−2.69	.008	−0.014	0.005	−0.201	−2.76	.006	−0.16	0.005	‐´232	2.91	.004	−0.013	0.005	−0.192	−2.67	.008
High educ^a^						0.467	0.160	0.222	2.91	.004											0.410	0.152	0.195	2.71	.008
Experience^a^											0.260	0.165	0.144	1.58	.117										
exp(good)^a^											0.485	0.157	0.283	3.09	.002						0.591	0.124	0.344	4.76	<.001
exp.(bad)^a^											−0.067	0.138	−0.036	−0.48	.631										
Work eng.																0.055	0.079	0.056	0.70	.487					
*R* ^2^ /adj,.*R* ^2^	0.046/0.040					0.091/0.080					0.187/0.166					0.052/0.040					0.211/0.195				
*F*	7.67 (*p* = .006)					7.85 (*p* = .001)					8.86 (*p* < .001)					4.24 (*p* = .016)					13.61 (*p* < .001)				

^a^ Dummy variable.

**FIGURE 1:**
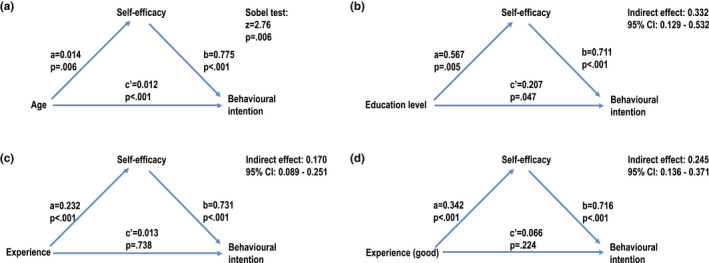
Direct and indirect effect of employees’ age (A), educational level (B) and previous experience with using welfare and health technology (C and D)

Mediation analysis reveals that age has both a direct effect (*p* < .001) and an indirect effect on behavioural intention, and self‐efficacy is the mediating variable here (‐z = 2.76, *p* = .006). The direct effect of age is positive, the indirect effect is negative, and the total effect is not significant when the effects cancel each other out (Figure 1).

### 5.5 Significance of educational level

According to the results, employees’ willingness to introduce robots depends on a person's educational level. Behavioural intention is significantly stronger, *t*(157) = 3.507, *p* = .001, in the group of graduates (*M* = 4.297, *SD* = 0.635) than among other employees (*M* = 3.757, *SD* = 0.822). However, the effect of educational level does not appear in the regression model 3 (Table 4). This is explained by the fact that self‐efficacy related to the use of robots, *t*(157) = 2.856, *p* = .005, is stronger in the group of graduates. According to regression analysis (Table 5), the effect of educational level on self‐efficacy is significant. Indeed, it seems that educational level has an indirect effect on behavioural intention through self‐efficacy. According to bootstrapping analysis, the indirect effect coefficient was 0.332 (95% CI = 0.129–0.532). Educational level has also little direct effect on behavioural intention (c´ = 0.207, *p* = .047), but the indirect effect is stronger (62% of total effect) (Figure 1).

### 5.6 Significance of employees’ previous experience with using welfare and health technology

Employees’ previous experience with using welfare and health technology is significantly associated with the willingness to introduce care robots, *r*_s_(158) = .275, *p* < .001, and self‐efficacy related to the use of care robots, *r*_s_(158) = .320, *p* < .001. Good experience with technology correlates significantly with behavioural intention, r_s_(158)=0.350, *p*<.001, and self‐efficacy, r_s_(158)=0.363, *p*<.001. In contrast, the existence of negative experiences does not correlate with behavioural intention, *r*_s_(158) = −.129, *p* = .103, or self‐efficacy, *r*_s_(158) = −.061, *p* = .441.

According to regression analysis, none of the dummy variables related to experience have a significant effect on the intention to introduce care robots (Table 4), and only the existence of good experiences has a significant effect on self‐efficacy (Table 5). The fact that the effect of the amount of experience on self‐efficacy is not significant in the regression model is explained by the strong interrelation between the amount of experience and the existence of good experiences, *r*_s_(158) = .610, *p* < .001.

It seems that previous experience with welfare and health technology has an indirect effect on the behavioural intention to introduce robots, and the effect is mediated through self‐efficacy. The indirect effect coefficient of previous experience was 0.170 (95% CI = 0.089–0.251), while the indirect effect coefficient of good experience was 0.245 (95% CI =0.136–0.371). Direct effects of previous experience (c´ = 0.013, *p* = .738) or good experience (c´ = 0.066, *p* = .224) are not significant (Figure 1).

### 5.7 Significance of work engagement

Work engagement does not correlate with behavioural intention, *r*(155) = .033, *p* = .685, or self‐efficacy, *r*(155) = .014, *p* = .866. According to regression analysis, work engagement does not explain variation in behavioural intention (Table 4) or self‐efficacy (Table 5).

## 6 DISCUSSION

The results are consistent with the theory of planned behaviour (Ajzen, 1991) and previous studies on the adoption of new technology in health care (i.e. Alaiad & Zhou, 2014; Holden & Karsh, 2010). The first hypothesis that “home care employees’ behavioural intention depends on self‐efficacy related to the use of care robots, attitudes towards the usefulness of robots in various home care tasks and perceived norms in the work community*”* is supposed. Consistent with previous studies, employees’ self‐efficacy (Rantanen et al., 2018; Latikka et al., 2019) and the social influence of familiar people (Alaiad & Zhou, 2014) are important factors in terms of the adoption of care robots in home care.

Previous studies on the effect of age on attitudes towards robots and the willingness to use robots have been contradictory (Flandorfer, 2012; Syrdal et al., 2013). The hypothesis that “employees’ age is associated with behavioural intention and self‐efficacy related to the use of care robots in home care” is supposed. However, the dependence is very complex. Ezer et al. (2009) has argued that the differences in age groups in the acceptance of robots are explained by previous experiences. This study refines this result and shows that differences in age groups are explained by self‐efficacy, and previous experiences, in turn, are related to self‐efficacy. This study shows that age reduces the sense of control related to the use of robots, but on the other hand, the ageing of the employee increases the willingness to use care robots in their work. Thus, nursing leaders should take into consideration that self‐efficacy is pivotal in the willingness to adopt care robots and that there are significant differences between age groups in self‐efficacy when they become involved in the introduction of robots (De Brún et al., 2019).

The third hypothesis “employees’ educational level and previous experience with using welfare and health technology are associated with behavioural intention and self‐efficacy related to the use of care robots in home care” is partially supposed. The finding is consistent with previous studies (Ezer et al., 2009; Flandorfer, 2012). However, the effect of cognitive factors is greater, and the effects of educational level and previous experiences on behavioural intention do not appear in a model that also includes variables describing cognitive factors. Employees with a bachelor's or master's degree have stronger confidence in their own technological competences, and through this, they have a stronger willingness to introduce and use care robots. Employees’ previous experience in health technology is relevant in terms of the introduction of care robots. It seems that positive experiences play a more important role than negative experiences. Thus, various pilot experiments in which employees gain experience can be considered important, and there is no need to fear possible failures.

In contrast, the hypothesis that “the level of employees’ work engagement is associated with behavioural intention and self‐efficacy related to the use of robots in home care*”* is not supposed. Employees’ general enthusiasm for the work is not connected to willingness to introduce robots. This is a surprise, as previous studies suggest that work engagement is linked to innovation (Schaufeli et al., 2006), and it could be assumed that vigour and innovation provide a starting point for job development through the introduction of robots.

### 6.1 Methodological strengths and limitations

The questionnaire was based on validated high‐reliability measures (α > 0.85). The response rate remained low (14.4%). However, representativeness was good. The data were collected in five locations across Finland. The distributions of gender, profession and region are close to national values. The study was based only on Finnish data, and so, direct comparison with other countries is not possible. People from different cultures have different attitudes towards robots and that should be considered when robotic products are being considered for certain countries (Papadopoulos and Koulouglioti, 2018).

## 7 CONCLUSION

It is notable that nurse leaders have an influential role in the implementation of new practices and in facilitating the willingness to adapt to new technologies by supporting technology use (Bianchi et al., 2018). Therefore, it is crucial for nursing leaders to take into consideration employees’ age, education and personal qualities, such as their level of self‐efficacy (Gifford et al., 2013). Lin (2016) noticed that the higher the general self‐efficacy and the self‐efficacy about nursing skills learning, the lower the anxiety levels towards nursing skills assessment subjects. Behavioural intention is significantly stronger in the group of graduates than among other employees. This is an important finding concerning home care nursing leadership because in Finland the majority of those who work in home care do not possess a higher education degree. The result is similar with previous studies, and there is a statistically high significant relation between nurses’ self‐efficacy and their age, occupation and educational level (Zaki, 2016).

## 8 IMPLICATIONS FOR NURSING MANAGEMENT

The introduction of robotics technology means changes to many work‐related practices, and thus, it is important to involve the entire work community. Our research recommends that it is also important to pay attention to supporting the employees’ sense of technology management and constructing a robot‐positive atmosphere when introducing care robots. The development of skills of older employees and employees with a lower level of education should also be supported. Change can be facilitated by various pilot projects where employees gain experience in adopting the technology. In contrast, the personnel's general enthusiasm for work, and support for it, is less central in this process of change.

## CONFLICT OF INTEREST

The authors declare no conflict of interest.

## Data Availability

Author elects to not share data.

## APPENDIX 1 Statements included in the measures

**Behavioural intention** (Likert scale).

Generally, I’m ready to and even enthusiastic about implementing new technological applications if they can enhance the quality of work of home care.

I would be ready for experimenting and introducing new robotic technology in home care if it could ease elderly people's independent coping at home.

I would be ready for the introduction of care robots that ensure the safety of elderly people who live at home.

I would be enthusiastic about introducing robotic technology that enhances the safety of medication in home care.

I would be very motivated to introducing robotic technology that eases everyday tasks in home care and to the instruction of clients it requires.

**Self‐efficacy** (Likert scale).

Generally speaking, I consider myself technologically competent.

I’m confident in my ability to learn how to use care robots, if they were to become part of my unit.

I believe that it would be easy for me to learn how to use the care robots that may be used in home care in the future.

I’m confident in my ability to learn simple programming of care robots if I were provided the necessary training.

I’m confident in my ability to learn how to use care robots to guide others to do the same.

I believe that teaching elderly people how to use care robots would not be difficult for me.

**Subjective norm** (Likert scale).

Generally, I’m ready to and even enthusiastic about implementing new technological applications if they can enhance the quality of work of home care.

My working community views the introduction of care robots for home care in a positive light.

My co‐workers are enthusiastic about the possible use of care robots in home care.

I believe that my working community would support the use of care robots in home care.

**Attitude towards care robots** (Likert scale).

A care robot can help an older person with washing, dressing up and using the toilet.

A care robot can help an older person move (e.g. moving from bed to chair).

A care robot can assist an older person in light household work (e.g. cooking, making their bed, doing the dishes, using the dishwasher).

A care robot can assist an older person in heavy household work (cleaning the windows, lifting).

A care robot can help an older person communicate with relatives and friends.

A care robot can help observing an older person's state of health (i.e. remote communication with doctor or nurse, real‐time conveying of health information).

A care robot can help with medication (e.g. giving medicine, recognising medicine, observing medicine use).

A care robot can contribute to the safety of an older person living at home (e.g. by informing relatives and/or healthcare workers of sudden change in the state of health).

A care robot can remind an older person to take their medicine, of week days, of meetings.

A care robot can guide an older person with the use of phone or bank‐related issues.

A care robot can guide an older person in physical exercise.

A care robot can ease the anxiety and loneliness of an elderly person.

A care robot can increase the independence of everyday life in older people.

A robot can increase the meaning of an elderly person's life.

**Work engagement** (Scale: Never, a few times a year, once a month, a few times a month, once a week, a few times a week, every day).

At my work, I feel bursting with energy.

At my job, I feel strong and vigorous.

I am enthusiastic about my job.

My job inspires me.

When I get up in the morning, I feel like going to work.

I feel happy when I am working intensely.

I am proud on the work that I do.

I am immersed in my work.

I get carried away when I’m working.

**Experience** (Likert scale).

I have a lot of experience with using welfare and healthcare technology.

**Experience (good)** (Likert scale).

I have good experience with using welfare and healthcare technology.

**Experience (bad)** (Likert scale).

I have experience that welfare and healthcare technology does not work as desired.
